# Nasal administration of anti-CD3 monoclonal antibody modulates effector CD8+ T cell function and induces a regulatory response in T cells in human subjects

**DOI:** 10.3389/fimmu.2022.956907

**Published:** 2022-11-23

**Authors:** Tanuja Chitnis, Belinda J. Kaskow, Junning Case, Katherine Hanus, Zhenhua Li, Johnna F. Varghese, Brian C. Healy, Christian Gauthier, Taylor J. Saraceno, Shrishti Saxena, Hrishikesh Lokhande, Thais G. Moreira, Jonathan Zurawski, Rachel E. Roditi, Regan W. Bergmark, Federico Giovannoni, Maria F. Torti, Zhaorong Li, Francisco Quintana, William A. Clementi, Kunwar Shailubhai, Howard L. Weiner, Clare M. Baecher-Allan

**Affiliations:** ^1^ Harvard Medical School, Boston, MA, United States; ^2^ Ann Romney Center for Neurologic Diseases, Department of Neurology, Brigham and Women’s Hospital, Boston, MA, United States; ^3^ Department of Otolaryngology-Head and Neck Surgery, Harvard Medical School, Boston, MA, United States; ^4^ Department of Surgery, Brigham and Women’s Hospital, Boston, MA, United States; ^5^ Clementi, Ltd., Rosemont, PA, United States; ^6^ Tiziana Life Science, Doylestown, PA, United States

**Keywords:** Foralumab, nasal anti-CD3, immunomodulation, CD8+ T cells, Tregs

## Abstract

**Background:**

Parenteral anti-CD3 Mab (OKT3) has been used to treat transplant rejection and parental administration of a humanized anti-CD3 Mab (Teplizumab) showed positive effects in diabetes. Nasal administration of anti-CD3 Mab has not been carried out in humans. Nasal anti-CD3 Mab suppresses autoimmune diseases and central nervous system (CNS) inflammation in animal models. We investigated the safety and immune effects of a fully humanized, previously uncharacterized nasal anti-CD3 Mab (Foralumab) in humans and its *in vitro* stimulatory properties.

**Methods:**

*In vitro*, Foralumab were compared to UCHT1 anti-human CD3 mAb. For human administration, 27 healthy volunteers (9 per group) received nasal Foralumab or placebo at a dose of 10ug, 50ug, or 250ug daily for 5 days. Safety was assessed and immune parameters measured on day 1 (pre-treatment), 7, 14, and 30 by FACS and by scRNAseq.

**Results:**

*In vitro*, Foralumab preferentially induced CD8+ T cell stimulation, reduced CD4+ T cell proliferation and lowered expression of IFNg, IL-17 and TNFa. Foralumab induced LAP, TIGIT, and KLRG1 immune checkpoint molecules on CD8+ and CD4+ T cells in a mechanism independent of CD8 T cells. *In vivo*, nasal Foralumab did not modulate CD3 from the T cell surface at any dose. Immune effects were primarily observed at the 50ug dose and consisted of reduction of CD8+ effector memory cells, an increase in naive CD8+ and CD4+ T cells, and reduced CD8+ T cell granzyme B and perforin expression. Differentially expressed genes observed by scRNAseq in CD8+ and CD4+ populations promoted survival and were anti-inflammatory. In the CD8+ TEMRA population there was induction of TIGIT, TGFB1 and KIR3DL2, indicative of a regulatory phenotype. In the memory CD4+ population, there was induction of CTLA4, KLRG1, and TGFB whereas there was an induction of TGF-B1 in naïve CD4+ T cells. In monocytes, there was induction of genes (HLA-DP, HLA-DQ) that promote a less inflammatory immune response. No side effects were observed, and no subjects developed human anti-mouse antibodies.

**Conclusion:**

These findings demonstrate that nasal Foralumab is safe and immunologically active in humans and presents a new avenue for the treatment of autoimmune and CNS diseases.

## Introduction

Intravenous CD3-specific monoclonal antibodies (Mab) have been investigated as a treatment for autoimmune and inflammatory disease both in mice and in humans (1). OKT3, a murine anti-CD3 Mab was FDA approved for the treatment of acute graft rejection in people (2) and was tested in an open label trial in subjects with Multiple Sclerosis (MS) (3). Because of side effects associated with IV Muramomab, primarily cytokine release syndrome (4) investigators engineered new generations of anti-human Fc-receptor non-binding CD3-specific antibodies for use in humans (1) and these have been tested in type 1 diabetes (5, 6) and inflammatory bowel disease (7, 8). One of these antibodies, Teplizumab has shown effectiveness in the treatment of type 1 diabetes (9).

A major challenge for the treatment of autoimmune and inflammatory diseases is the induction of an anti-inflammatory response that ameliorates disease with minimal side effects. One such mechanism is the induction of regulatory T cells which have been shown to play a major role in both animal models and human diseases (10). The mucosal immune system, which includes both the gastrointestinal tract and the nasal cavity, has a well-developed immune response by which the organism interfaces with the external environment and plays a key role in immune homeostasis. It has long been recognized that immune interactions at mucosal surfaces induce tolerogenic responses, of which the induction of regulatory T cells is a major component. The induction of regulatory cells at mucosal surfaces by the oral or nasal administration of antigen has been shown to treat a large variety of autoimmune and inflammatory diseases in animal models (11). In addition, we found that the mucosal administration of anti-CD3 mAb by binding to the T cell receptor in the mucosal environment induces regulatory IL-10-producing T cells and treats autoimmune and inflammatory diseases including models of MS (10, 12), diabetes (1, 13, 14), arthritis (15), and lupus (16). Others have shown that oral anti-CD3 treats models of colitis (17) and atherosclerosis (18).

In human trials that we and others have conducted, oral OKT3 was administered to healthy volunteers (19) and to subjects with nonalcoholic steatohepatitis (20), chronic hepatitis C infection (21), and ulcerative colitis (22). In these pilot trials, oral OKT3 induced immune effects and was well tolerated. Because of our interest in the treatment of progressive MS, because we found that nasal but not oral anti-CD3 mAb ameliorated disease in a progressive animal model of MS (10) and because anti-CD3 mAb had never been given before to humans, we initiated a dosing and safety study of nasal anti-CD3 in healthy human volunteers. We chose Foralumab a fully human anti-CD3 monoclonal antibody that has been given by the intravenous route to patients with moderate to severe active Crohn’s disease (8). Foralumab has a mutated IgG1arm designed to reduce the binding to Fc gamma receptors to minimize infusion related reactions associated with cytokine release when it is given intravenously. IV Foralumab was given for 5 days in a dose escalation study of 0.05 mg (n = 5); 0.1mg (n = 3); 0.5 mg (n = 11); 1 mg (n = 12), and 2 mg (n = 1) in patients with moderate to severe active Crohn’s disease (8). Due to the side effects in the patient receiving 2 mg, the dose was not escalated above 1 mg. Our goal was to evaluate the safety and immune responses of nasally administered Foralumab. We performed a dose-escalation study of Foralumab in healthy subjects at three dose-levels (10 µg, 50 µg, and 250 µg) administered intranasally daily for 5 days to assess safety, and to determine which, if any, dose induced immune responses in humans. We found that nasal Foralumab was safe and induced immune responses at the 50ug dose that included modulation of effector CD8+ T cell function and induced a regulatory T cell response.

## Methods

### Subjects and study design

The study was a randomized, double blind dose escalation study of 10μg, 50μg or 250μg nasal Foralumab given for 5 days (n=6) or placebo (n=3) at each dose level. Placebo consisted of phosphate acetate buffer. One spray was given into each nostril. There were two sentinel subjects at each dose level (one placebo and one active treatment) to evaluate for serious adverse events. Each subject participated for 30 days. Participants were healthy volunteers, women and men ages 18 to 65 participated. All subjects underwent informed consent and were treated at the Brigham and Women’s Hospital’s Center for Clinical Investigation (CCI). A controlled particle dispersion device from Kurve Technology^®^ was used for nasal delivery of Foralumab. Patients signed an informed consent form. The study was approved by the Mass General Brigham Human Subjects Research Committee (IRB).

### Study drug

Foralumab (28F11-AE; NI-0401) is a fully human IgG1 anti-CD3 mAb with the Fc portion mutated such that the mAb is non FcR binding *in vitro* which exhibits only minor cytokine release *in vivo* while maintaining modulation of the CD3/TCR and T cell depletion when given I.V (8, 23). Foralumab was developed by Novimmune and was acquired by Tiziana Life Sciences.

### Clinical and laboratory evaluation

Subjects underwent clinical (vital signs) and laboratory evaluation (hematology, serum chemistry and urinalysis) for safety and adverse events at days 7, 15 and 30 at which time blood was drawn for immunologic studies. An otolaryngology physical exam including sinonasal endoscopy was performed by an otolaryngologist at the screening visit, visit 5 (final dosing day), and at visit 9 (day 30). A nasal questionnaire was administered at all visits throughout the study.

### Analysis of *In vitro* T cell stimulation by Foralumab using healthy donor PBMCs

Various T cell populations were isolated from cryopreserved healthy donor ficoll-gradient-isolated, PBMCs. Total T cells were isolated using the human Pan T cell isolation kit (Miltenyi Biotec), CD4 T cells were isolated using the human CD4 T cell isolation kit (Miltenyi Biotec) or were FACS-sorted to give rise to total CD4 and non-Treg CD4 T cells, and CD8 T cells were FACS-sorted from total PBMCs. For FACS sorting, the PBMCs were labeled with e506 viability dye (ThermoFisher), incubated with FcR block, and stained with CD4 (RPA-T4), CD25 (BC96), CD127 (A019D5), CD8 (RPA-T8), and CD14 (M5E2) from BioLegend. Autologous accessory cells were isolated as either irradiated total PBMCs or CD3-depleted (Miltenyi Biotec) irradiated PBMCs (3,000 rad). Total T cells, CD4 T cells, and non-regulatory CD4 T cells were labelled with Cell Trace Violet (CTV), while the isolated CD8 T cells were labelled with Cell Trace Far Red (CTFR) (ThermoFisher). The different T cells and autologous accessory cells were stimulated *in vitro* with soluble anti-CD3 Abs (UCHT1 or Foralumab, 2ug/ml) and rhIL-2 (5U/ml) or with increasing anti-CD3 doses from 1ug/ml to 8ug/ml, all with rhIL-2 (5U/ml). Replicate control cultures were established that contained no anti-CD3 but were given either PBS or isotypes at 2ug/ml (mouse IgG1, and human IgG1), which produced the same lack of stimulation. Cultures were established with different T cells at the indicated cell number per well: 5x10^3^ “All” (Pan) T cells, 5x10^3^ CD4 (CTV) T cells, 5x10^3^ CD8 (CTFR) T cells, or combined 3.3 x10^3^ CD4 (CTV) and 1.67x10^3^ CD8 (CTFR) T cells (2:1 ratio of CD4: CD8). All cultures received 1x10^4^ autologous APCs. Cultures were established in a minimum of triplicate wells in 96-well U-bottom plates (Costar) in RPMI-1640 medium (Life Technologies) supplemented with Na Pyruvate, NEAA, HEPES, Glutamine and PennStrep (all from Gibco), and 2% HuS (Gemini Bioproducts). After 5 days, the cultures were stained for viability, stimulated with PMA/Ionomycin and golgistop (BD activation cocktail), and stained for surface CD8 (RPA-T8 from BD), and CD4 (OKT4), TIGIT (VSTM3), KLRG1 (MAFA), and LAP (TW4-2F8), then after fixation/permeabilization with intracellular IFNg (4S.B3), IL-17 (BL168), TNFa (MAB11) and FoxP3 (206D) (all from Biolegend) using the FoxP3 buffer set from BioLegend. The samples were run on a BD Fortessa FACS Analyzer and analyzed *via* Flowjo (BD) and prism software (graphing and statistics).

### Blood sample processing

PBMC samples used in the *in vitro* analysis of Foralumab activity were obtained from healthy donors who donated blood under our IRB approved for human studies. Trial participant subjects gave blood samples at baseline (T1) and at visits scheduled for 7 (T2), 14 (T3), and 28 (T4) days after drug administration under a separate approved IRB. The dates of follow-up varied slightly. Thus, T2 was at 7-10 days, T3 was at 14-18 days, and T4 was at 25-34 days. All blood samples were processed immediately. Plasma was removed by centrifugation of the sodium heparin blood collection tubes after which the blood was then resuspended, diluted with PBS at 1:1 ratio and applied to Ficoll-Hypaque (GE Healthcare) centrifugation to isolate the PBMC buffy coat. PBMCs were counted and resuspended in freezing media (90% FBS/10%DMSO) at 2x10^7^ PBMCs/vial and cryopreserved in liquid nitrogen.

### Analysis of trial participant longitudinal PBMCs by flow cytometry

PBMCs were thawed at 37C into complete RPMI media (with 2% Human AB serum, Gemini Bio), washed with PBS and stained for viability (eFluor 506 viability dye, Invitrogen). 5x10^6^ cells from each sample were subjected to surface stain for lineage and maturation markers followed by staining for intracellular proteins GzmB, Perf, and FoxP3. For surface staining, the cells were resuspended in FcR block (30% in MACS buffer for 15’ at 4C), and then incubated (40’ at 4C) with the panel of surface antibodies that included CD19 (LT19, Miltenyi Biotec), antibodies from Biolegend: CD3 (SK7), CD45RA (HI100), CD127 (A019D5), CD56 (NC1M16.2), CD20 (2H7), and LAP (TW4-6H10); antibodies from BD Bioscience: CD4 (SK3), CD8 (SK1), and CD27 (M-T271). After washing with MACs Buffer (0.1%FBS/PBS, 4C), cells were fixed and permeabilized using the eBioScience FoxP3 fixation buffer set, then incubated with permeabilization buffer containing 10% NRS (normal rat serum, 10’ at 4C), followed by incubation (30’ at 4C) with a panel of intracellular antibodies that included antibodies from Biolegend: Ki67 (KI67), FoxP3 (206D), IFNg (4S.B3), IL-17 (BL168), IL-10 (JES3-9D7) and Perf (dG9), and GzmB (GB11, BD Bioscience). The samples were washed with MACS buffer and each entire sample analyzed on a BD FACS Symphony flow cytometer with HTS attachment.

### Statistical analysis of the flow cytometric data

For comparison of change with time for each of the 57 immunologic markers, each treatment group (10ug, 50ug and 250ug groups and combined placebo patients) were analyzed separately. In each group, the change with time was estimated using a linear mixed effects model with a fixed categorical effect of time and a random intercept. The categorical effect of time allows estimation of the change from the first measurement to each of the subsequent measurements. The random intercept was included to account for the within patient correlation. Subjects with missing measurements were included in this analysis.

### Single cell RNAseq

Immune cells from the participants that received 50ug Foralumab were analyzed by scRNA-Seq using the 10X Genomics platform. Specific immune populations (CD4^+^ T cells, CD8^+^T cells, FoxP3^+^ Tregs, B cells, monocytes and dendritic cells), were FACS-sorted from the T1-T4 PBMCs, hash-tagged, and combined to generate specific samples (see **Supplementary Figure 1** for the FACS-sort gating and antibodies). All samples were submitted and processed through 10X Genomics CellRanger pipeline (v3.0). The analysis of the resultant filtered count matrices was conducted using the Seurat single cell toolkit (v4.1) in R. Count matrices were first demultiplexed and filtered to remove any doublets and negatives. Demultiplexed samples were then filtered further to remove cells with high mitochondrial gene transcript percentages (>20%), cells with low feature diversity (<1000 UMIs), and cells with abnormally high transcript counts (>20000). Data was then normalized and scaled by using Seurat’s default parameters with NormalizeData, FindVariableFeatures, and ScaleData functions. PCA was used to reduce the dimensions of the dataset before clustering the cells. Visualization of the clustering was completed through use of the UMAP algorithm packaged within Seurat. Removal of unwanted influence of gender differences was completed using the Harmony package (v0.1.0) before running differential expression analysis within Seurat. Accessory packages for the analysis and visualization of results were dittoSeq (v1.4.4) and ggplot2 (v3.3.5).

### Antigen arrays

Antigens were transferred to 384-well polypropylene plates (Genetix, X6004), resuspended in DMSO (1mg/mL) and spotted onto Epoxy microarray slides (Grace Bio-Labs, 405278) using a microarrayer (Aushon 2470) equipped with solid spotting pins. The microarrays slides were then blocked for 1 h at 37°C with 1% BSA and incubated for 2 h at 37°C with a 1:10 dilution of the samples in blocking buffer. The slides were later washed and incubated for 1 h at 37°C with a 1:100 dilution of goat anti-human IgG Cy3-conjugated and goat anti-human IgM AF647-conjugated detection antibodies (Jackson ImmunoResearch). Blocking, probing, and washing steps were performed using an HS 4800 Pro Hybridization Station (Tecan). Finally, the slides were scanned using a microarray scanner (Tecan Powerscanner).

### Statistical analysis of antigen array data

Statistical analysis was performed on the normalized intensities. The p values were obtained by using the generalized linear model in R and the log2 fold change was obtained by log transforming the ratio between the medians of two conditions.

## Results

### Demographics and study outline

The demographic characteristics of each cohort (10ug, 50ug, and 250ug) and the placebo are shown in **Table 1**. The patient disposition is shown in **Supplemental Figure 2**. One patient in each dosing cohort discontinued in the study due to non-drug related reasons.

**Table 1:** Demographics of nasal Foralumab dose cohorts (10μg, 50μg, 250μg) and placebo group.

	Placebo	Foralumab (10μg)	Foralumab (50μg)	Foralumab (250μg)	Foralumab-all doses
N	9	6	6	6	18
Male	6 (66.7%)	4 (66.7%)	3 (50%)	1 (16.7%)	8 (44.4%)
Female	3 (33.3%)	2 (33.3%)	3 (50%)	5 (83.3%)	10 (55.6%)
Mean age +/-SD (range) in years	38.0+/- 16.6 (22-65)	35.8+/-15.5 (23-60)	24.3+/-3.4 (19-29)	29.5+/-11.0 (21-51)	29.9+/- 11.5 (19-60)
Race					
White	6 (66.7%)	3 (50%)	2 (33.3%)	1 (16.7%)	6 (33.3%)
Black or African American	2 (22.2%)	0 (0%)	0 (0%)	1 (16.7%)	1 (5.6%)
Asian	1 (11.1%)	1 (16.7%)	2 (33.3%)	2 (33.3%)	5 (27.8%)
American Indian or Alaska Native	0 (0%)	0 (0%)	0 (0%)	0 (0%)	0 (0%)
Native Hawaiian or other Pacific	0 (0%)	0 (0%)	0 (0%)	0 (0%)	0 (0%)
Other or Unknown ethinicity	0 (0%)	2 (33.3%)	2 (33.3%)	2 (33.3%)	6 (33.3%)
Hispanic or Latino	1 (11.1%)	1 (16.7%)	2 (33.3%)	1 (16.7%)	4 (22.2%)
Non-hispanic or Latino	8 (88.9%)	3 (50%)	4 (66.7%)	5 (83.3%)	12 (66.7%)
Unknown or not reported	0 (0%)	2 (33.3%)	0 (0%)	0 (0%)	2 (11.1%)

### Safety

The treatment was well tolerated by all subjects. No systemic effects were observed at any dose including changes in vital signs (temperature, pulse, blood pressure) or in liver, kidney and hematologic measures (complete blood counts, including differential) during treatment or follow-up. No abnormalities were observed on otolaryngology examination. No EBV reactivation was observed (**Table 2**).

**Table 2:** Treatment-emergent adverse event (TEAE).

System organ class	PlaceboN=9	Foralumab (10ug) N=6	Foralumab (50ug) N=6	Foralumab (250ug) N=6	Foralumab-all doses N=18
**Subjects with at least one TEAE**	2	5	5	2	12
**Number of TEAEs by severity**	3	15	9	3	27
**Severity**					**27**
Non-serious	3	15	9	3	27
Serious	0	0	0	0	0
**Ear and labyrinth disorders**					**3**
Non-serious	0	2	1	0	3
Serious	0	0	0	0	0
**Gastrointestinal disorders**					**2**
Non-serious	0	0	2	0	2
Serious	0	0	0	0	0
**Immune system disorder**					**1**
Non-serious	0	0	1	0	1
Serious	0	0	0	0	0
**Infections and infestations**					**9**
Non-serious	0	8	0	1	9
Serious	0	0	0	0	0
**Injury, poisoning and procedural complications**					**1**
Non-serious	0	1	0	0	1
Serious	0	0	0	0	0
**Nervous system disorders**					**6**
Non-serious	0	3	2	1	6
Serious	0	0	0	0	0
**Skin and subcutaneous tissue disorder**					**5**
Non-serious	3	1	3	1	5
Serious	0	0	0	0	0

Bold values are p<0.05.

### Unique *In vitro* T cell activation by Foralumab

**Figure 1:**
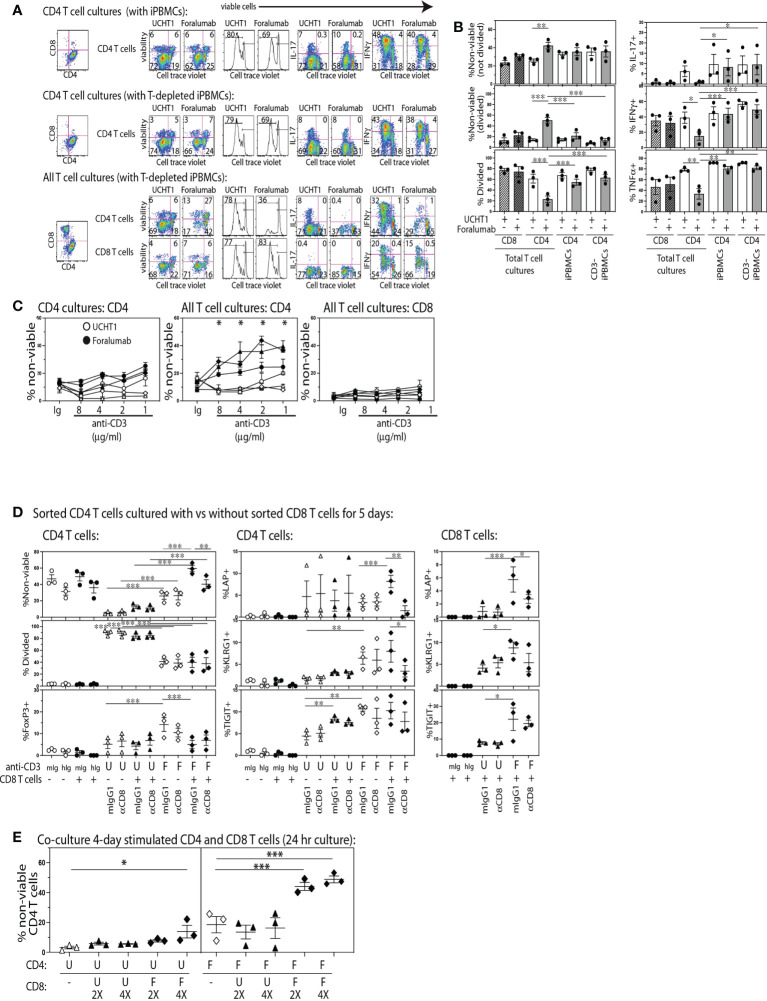
The Foralumab anti-CD3 mAb exhibits unique *in vitro* stimulation properties *via* its capacity to induce regulatory function in CD8 T cells. **(A)** Representative FACs plots are shown for the changes in viability, proliferation and cytokine expression by cell trace labeled CD4 T cells when stimulated with UCHT1/IL2 or Foralumab/IL2 when purified or in the presence of non-CD4 T cells, and irradiated PBMCs (with and without T cell depletion) as APCs. **(B)** Graphical analysis of changes in features within the cell trace diluted CD4 T cells (from three healthy donors) in response to the different culture conditions. **(C)** Changes in viability was examined in a dose response of 5,000 T cells stimulated with Foralumab/IL2 (filled) vs UCHT1/IL-2 (open) as compared to mIgG1/IL-2 or huIgG1/IL-2. Viability was determined at 5 days by e506 staining. **(D)** FACS-sorted total CD4 T cells were stimulated alone (open) or together with autologous FACS-sorted CD8 T cells (filled) with mIgG1 (isotype for UCHT1), huIgG1 (isotype for Foralumab), UCHT1 or Foralumab. All in the presence of IL-2 (5U/ml), and T cell depleted irradiated PBMCS as APCs. On day 5, the cultures were harvested and measured for viability, proliferation, and surface expression of LAP, KLRG1 and TIGIT, and intracellular FoxP3. **(E)** CD8 T cells that were stimulated with Foralumab for 4 days were tested for the capacity to kill activated UCHT1 or Foralumab stimulated CD4 T cells. On day 4, the different anti-CD3 stimulated CD4 and CD8 T cells were harvested, washed and re-plated in all combinations in the presence of IL-2 (5U/ml) at the indicated ratios (5,000 CD4 and 2X or 4X activated CD8 T cells). The information for the isotypes reflected features expressed by cell trace high populations as there was no proliferation. For all graphs, significance was determined by One-Way ANOVA with Sidak’s correction for multiple comparisons, * p<0.05, **p<0.01, ***p<0.005.

To determine if stimulation through the fully human, FcR modified, Foralumab antibody gave unbiased human T cell proliferation analogous to that induced by other anti-CD3 antibodies commonly used in research, we established replicate cultures of CD4 T cells (CTV) stimulated with APCs consisting of either total or CD3-depleted PBMCs, and cultures of All T cells (CTV) (containing both CD4 and CD8 from Pan T cell isolation) stimulated with T cell depleted PBMCs. The cultures were stimulated with either Foralumab (modified IgG1) or the UCHT1 (IgG1) anti-CD3 mAb. After 5 days, the cultures were stained to determine viability, CD4^+^ and CD8^+^ lineage, extent of proliferation using cell trace dilution, and cytokine expression. As shown in **Figure 1** we found that Foralumab induced strikingly lower CD4 T cell proliferation in cultures established with All T cells as compared to UCHT1 stimulation (**Figures 1A, B**). The reduced proliferation of the CD4 T cells in the ‘All T cell’ cultures was also associated with a significant increase in loss of viability by both divided and non-divided CD4 T cells. As poor TCR signaling could give rise to low viability, we tested whether the poor viability of CD4 T cells in Foralumab stimulated All T cell cultures was abrogated by increasing the dose of the Foralumab Mab (**Figure 1C**). Upon establishing the assay with increasing amounts of UCHT1 or Foralumab in cultures of either purified CD4 T cells vs all T cells, the data suggests that competition for anti-CD3 Mab does not affect the outcome as poor CD4 T cell viability was observed at all doses of Foralumab as compared to UCHT1 cultures, and only in the cultures that contained all T cells.

Inhibitory effects of anti-CD3 in humans have been proposed to act by altering the balance of Th subsets (24, 25). Thus, we also examined whether Foralumab stimulation resulted in an altered Th1 or Th17 frequency. As shown in **Figures 1A**, **B**, we found that in the presence of CD8^+^ T cells, Foralumab stimulated CD4^+^ T cells exhibited reduced expression of IFNg (Th1), IL-17 (Th17) and TNFa as compared to the Foralumab or UCHT1 stimulation of pure CD4^+^ T cells. In contrast the CD8 T cells in these cultures showed no change in viability, proliferation or frequency of cytokine expression when stimulated with UCHT1 or Foralumab.

We next asked specifically whether CD8 T cells had to be present for Foralumab to exert these unusual effects on CD4 T cell viability and expansion by establishing cultures of FACS-sorted CD4 T cells with and without autologous FACS-sorted CD8 T cells and stimulated with either anti-CD3 Mab (**Figure 1D**). In addition, in order to test the potential mechanism whereby the CD8 T cells regulate the co-cultured CD4 T cells, replicate assays were also established in the presence or absence (isotype) of a blocking anti-CD8 Mab (SL1) known to reduce CD8 T tetramer binding and activation (26, 27). In these assays, as compared to UCHT1 stimulation, while CD4 T cells stimulated alone with Foralumab showed some reduction in viability and proliferation, if the cultures contained CD8 T cells there was an extremely high loss of CD4 T cell viability that was sensitive to reversal with CD8 blockade. In addition, Foralumab stimulation markedly induced the expression of LAP, KLRG1 and TIGIT on CD4 T cells as well as on the CD8 T cells in cultures where they had been added. These data suggest that Foralumab may directly induce greater inhibitory molecules on CD8 T cells and on CD4 T cells stimulated in the presence and absence of CD8 T cells. Of note, the blockade of CD8, which is known to reduce CD8 activation, also reduced the CD8 T cell expression of LAP. Yet, although these data suggest that Foralumab induced a CD8^+^ T cell-mediated regulation of CD4^+^ T cells, we did not observe an increase in FoxP3 expression in Foralumab-stimulated CD8 T cells in these cultures (data not shown). Similar results were obtained when this assay was replicated with CD4 T cells that were FACS sorted to lack CD25^hi^CD127^lo^ regulatory T cells (**Supplementary Figure 3**). Additional assays were established to address whether Foralumab mediated regulation of CD4 T cell activation arose due to nutrient exhaustion. Although the assays were only established with 5,000 T cells, we excluded the potential role of nutrient exhaustion by replacing media at day 3 in one set of replicate plates vs the control set of cultures that remained untouched until day 5 (see **Supplementary Figure 4**). All together these data suggest that Foralumab-stimulation induces CD8 T cells to exhibit a CD4 killing/regulatory function.

Although identifying an anti-TCR mAb that selectively activates CD8^+^ vs CD4^+^ T cells might appear unusual, the humanized, Fc-altered Teplizumab anti-CD3 mAb, was also reported to induce selective *in vitro* expansion of CD8^+^ T cells (28), which they proposed arose due to Teplizumab inducing a population of CD8^+^FoxP3^+^ regulatory T cells that killed the CD4^+^ T cells that were present in the same culture. Thus, we next asked whether Foralumab acted *via* this mechanism of inducing regulatory CD8+T cells. To test this, we separately stimulated FACS purified CD4 and CD8 T cells with Foralumab or UCHT1 for four days, before the cells were harvested, washed and re-plated in all combination overnight co-cultures at CD4:CD8 ratios of 1:0, 1:2, and 1:4. As shown in **Figure 1E**, the Foralumab stimulated CD8 T cells not only induced a striking loss of viability by Foralumab-stimulated CD4 T cells, but also reduced the viability of the UCHT1 stimulated CD4 T cells at the 1:4 ratio. Although these data strongly suggest that Foralumab induces regulatory function in CD8 T cells (29) and also increases the sensitivity of CD4 T cells to regulation, the mechanism by which Foralumab exerts this unusual activity remains unclear.

### Nasal Foralumab does not modulate CD3 from the T cell surface

**Figure 2:**
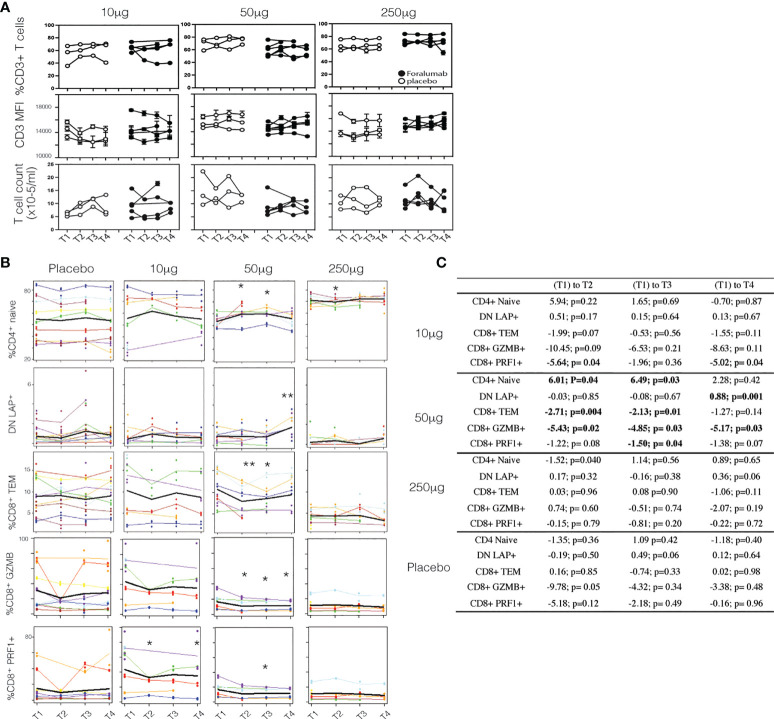
Surface CD3 and Foralumab dosing **(A)** Longitudinal PBMCs were stained for CD3 and measured for changes in frequency of CD3+ cells, the intensity of CD3 (MFI) and total T cell counts as compared to baseline (T1) levels. **(B)** Lineage and differentiation markers in 10ug, 50ug, 250ug and placebo groups. CD4+CD45RA+CD27+ are CD4+ naïve; CD3+CD4-CD8- are DN LAP+; CD8+CD45RA-CD27- CD8 Tem; GZMB+ and PRF1+ in total CD8+ cells is shown. **(C)** Shows the change with time was estimated using a linear mixed effects model with a fixed categorical effect of time and a random intercept. *p<0.05, **p<0.001). N=6, placebo N=6.

IV administration of anti-CD3 mAbs induces the down-modulation of CD3 from the T cell surface (1). In the study of IV Foralumab in Crohn’s disease, CD3 modulation was observed at all dose levels (50ug, 100ug, 500ug and 1000ug) with the greatest effect seen at the 500 ug and 1000 ug doses. The highest dose of Foralumab we administered nasally was 250ug which is generally less than what has been administered IV with Foralumab and other mAbs (1). In animal studies, we did not observe downregulation of CD3 on T cells following oral (12) or nasal administration of anti-CD3 even at doses that resulted in modulation of CD3 given by the IV route. Whether the lower amounts of Foralumab and the nasal route of administration would result in modulation of cell surface CD3 is unknown. To address this, we stained the longitudinal PBMC samples from baseline (T1) and the T2, T3, and T4 timepoints after the 5-day regimen of daily nasal Foralumab and performed cytometric analysis to determine the frequency and mean fluorescence intensity of the cells that bound anti-CD3, and whether the number of circulating CD3+ cells change overtime with treatment. As shown in **Figure 2A**, we found no change in the frequency of CD3^+^ cells (top) or intensity of CD3 expression (MFI, middle) as well as T cell counts at any dose in samples obtained beginning at 3 days after the dosing (T2). We also found no change in the frequencies of B cells (as percent of PBMC) or FoxP3^+^ Tregs (as percent of CD4 T cells) or in the CD4 and CD8 ratios over time (not shown).

### Immune effects of nasal Foralumab occur at the 50ug dose

To determine whether immunologic effects were observed following nasal Foralumab, we stimulated PBMCs with PMA/ionomycin for 4 hours and then stained by flow cytometry for surface and intracellular proteins. We compared pre-treatment (T1) vs the post treatment (T2, T3, and T4) timepoints for the 10ug, 50ug, and 250ug doses and placebo. We found reductions in pro-inflammatory, activated subsets of both CD4 and CD8 T cells that were primarily observed in the group that received the 50 ug dose. We used CD27 expression in lieu of CCR7 to define maturational states as CCR7 expression is reduced on T cells after cryogenic preservation (30). In terms of CD8+ cells, as shown in **Figures 2B, C**, there was a decrease in the frequency of effector memory cells at T2 and T3. Additional changes observed in CD8^+^ cells in the 50ug dose included decreased frequency of TEMRAs (CD45RA^+^CD27^-^), increased frequency of naïve cells (CD45RA^+^CD27^+^) (**Figures 2B, C**), and decreased expression of granzyme B and Perforin 1, though no changes were observed in CD8^+^ central memory cells (**Figures 2B, C**; **Table 3**). In terms of CD4+ cells, as shown in **Figures 2B, C** we found an increase in CD4^+^ naïve cells at time point 2 and 3. As shown in **Table 3** other changes were observed in CD4^+^ cells at the 50ug dose including a decrease in frequency of CD4^+^ effector memory (Tem,CD3^+^CD4^+^CD45RA^-^CD27^-^) and TEMRAs (CD3^+^CD4^+^CD45RA^+^CD27^-^) as well as a decrease in granzyme B expression in CD4+ cells. As was the case with CD8^+^ cells, no changes were observed in CD4^+^ central memory cells. No changes were observed in CD4^+^ Foxp3^+^ cells. No other changes were observed. Of note at the 10ug dose we observed a decrease in CD8 perforin (T2 and T4 (**Figures 2B, C**). The lower number of changes observed at the 250ug dose is consistent observations that immunomodulatory immune effects may be lost at higher doses (11, 31).

**Table 3:** Estimated change from baseline to each follow-up time point in patients in the 50 μg dose.

	(T1) to T2	(T1) to T3	(T1) to T4
CD4 FoxP3+	0.16; p=0.61	-0.01; p=0.98	0.16; p=0.63	
CD8+nv	**8.07; p=0.003**	**6.75; p=0.01**	**5.69; p=0.03**	
CD8+GzmB+	**-5.43; p=0.02**	**-4.85; p=0.03**	**-5.17; p=0.03**	
CD8+Tem	**-2.71; p=0.004**	**-2.13; p=0.01**	-1.27; p=0.14	
CD8+TEMRA	**-4.01; p=0.04**	-3.8; p=0.06	**-4.4; p=0.04**	
CD8+Prf+	-1.22; p=0.08	**-1.5; p=0.04**	-1.38; p=0.07	
CD8+Tcm	-1.44; p=0.20	-1.22; p=0.27	-0.6; p=0.61	
CD4+nv	**6.01; p=0.04**	**6.49; p=0.03**	2.28; p=0.42	
CD4+Tem	-3.1; p=0.07	**-3.29; p=0.05**	-0.66; p=0.70	
CD4+TEMRA	**-2.24; p=0.05**	**-2.26; p=0.05**	-1.81; p=0.13	
CD4+GzmB+	-2.05; p=0.06	**-2.25; p=0.04**	-1.57; p=0.17	
CD4+Tcm	-0.91; p=0.51	-0.84; p=0.54	0.03; p=0.98	

The estimated difference and p-value were calculated using a mixed effects model with a random intercept. Positive estimated differences indicate that the mean level of the marker increased after administration of the treatment. Bold entries had a p-value less than 0.05.

### ScRNAseq analysis in subjects receiving the 50ug dose

**Figure 3:**
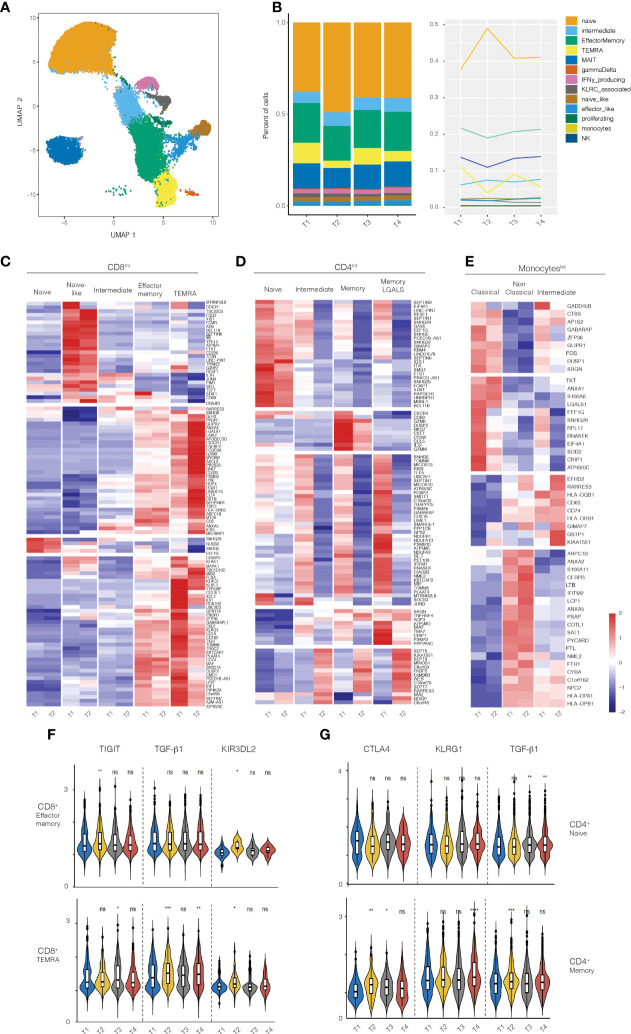
RNA-seq analysis on PBMCs from healthy volunteers treated with 50ug of Foralumab. **(A)** Graphical depiction of the single cell analysis of the CD8+ population isolated from PBMCs showing the cell types that were defined by the clusters based on unbiased DEG the changes in CD8 maturational state subsets derived from the scRNA data in aggregated bar **(B)** graphs or line graph analyses. **(C)** Different maturational subsets of CD8 T cells show unique DEG between baseline (T1) and T2. Heatmap presentation of the genes that exhibited increased or decreased expression from baseline after Foralumab treatment. Gene expression values were used to separate the cells into naïve CD8 T cells, naïve-like CD8 T cells (differed from naïve in expression of top group of genes), intermediate CD8 T cells (exhibited features of both naïve and memory cells), effector memory, and TEMRA. **(D)** Different maturational subsets of CD4 T cells Gene expression values were used to separate the cells into naïve CD4 T cells, intermediate CD4 T cells (exhibited features of both naïve and memory cells), memory CD4, and memory CD4 with strong GALS1 gene expression. **(E)** Different functional subsets of monocytes. Gene expression values were used to separate the cells into classical, non-classical and intermediate subsets. **(F)** Violin plots showing changes in expression of TIGIT, TGFb1 and KIR3DL2 in CD8 effector memory and CD8 TEMRA cells. **(G)** Violin plots showing changes in expression of CTLA4, KLRG1, and TGFb1 in naïve CD4 T cells and memory CD4 T cells. Unpaired two-sided T-test against timepoint 1 (T1vT2, T1vT3, T1vT4) was used to get the posted significance score if it changed from the baseline. *p<0.05, **p<0.001, ***p<0.0001, ****P<0.0001, ns, not significant).

Given that immunologic effects were primarily observed in subjects receiving the 50ug dose, we performed scRNAseq on isolated immune populations at baseline and post-treatment. Cell populations were FACS-sorted at the same time to prevent batch effects. Consistent with the flow cytometry analysis above, scRNAseq analysis showed a decrease in the frequency CD8 TEMRA and effector memory cells and an increase in the frequency of naïve CD8^+^ T cells (**Figure 3**). Most of the changes were observed in the first timepoint after 5 days of treatment (T1, baseline vs T2).

We then identified genes that were differentially expressed (DEGs) between baseline (T1) and 3-5 days after treatment (T2) in FACS-sorted CD8^+^, CD4^+^, Treg, and monocyte populations (**Figures 3C–E**). CD8^+^ T cells exhibited the highest number of DEG (109 genes), with CD4 T cells (non-regulatory), Tregs, and monocytes exhibiting DEG in 94 genes, 5 genes, and 3 genes respectively. Some of the DEG functioned in homeostatic cell biological processes (CD8-28%, CD4-53%, Treg-20%, monocyte-33%), whereas most up- or down-regulated genes have immunologic functions (78 genes in CD8 T cells, 44 genes in CD4 T cells, 4 genes in Tregs, and 2 genes in monocytes).

We then examined the function of the immune-related DEG in each cell type to elucidate the immune pathways affected by nasal Foralumab. In the CD8^+^ T cells (**Figure 3C**), the genes that were down regulated were primarily involved in promoting survival (STAT1, MTRNR2L8, PIM1, FCMR, and IL7R), augmenting cytokine production (BCL11B, ETS1, TRIM22, TDF7 and JUNB), inducing cell dysfunction (CD160, DUSP2), and enhancing cytotoxicity/signaling (PIP4K2A, PIK3R1, XCL1, FLNA, KLRF1, KLRK, and MAPK1). In contrast, the genes that were upregulated in CD8 T cells were anti-inflammatory as they limit protease/proteosome activity (RARRES3, PSMB2), augment anti-oxidative defense (GLRX, IERS), increase expression of inhibitory receptors (LAIR2, LY6E, and AXNA5), and yet also may promote migration (CX3CR1 and ITGB1).

We then asked whether the response of memory CD8^+^ T cell subsets to nasal Foralumab included the induction of TIGIT which is associated with the IV administered anti-CD3 antibody Teplizumab (32) that has efficacy in treating T1D patients (9) and the induction of certain KIR family member genes which have recently been shown to play a role in regulating autoimmune responses (33). Indeed, as shown in **Figure 3F**, we found that the nasal Foralumab treated CD8^+^effector memory populations showed induction of TIGIT (T2), and KIR3DL2 (T2) whereas the CD8+ TEMRA population exhibited induction of TIGIT (T3), TGF-B1 (T2,T4) and KIR3DL2 (T2).

The scRNA-Seq analysis of the non-regulatory CD4^+^ T cells indicated that Foralumab treatment resulted in reduced gene expression by all maturational subsets (**Figure 3D**). The most affected CD4^+^ T cells were in the activated subsets (intermediate, memory and LGALS1 signature cells) which exhibited reduced expression of genes involved in promoting cell migration (CXCR4, NKG7, CCL5, GzmM and SRGN), cytokine production/signaling (ETS1, IL6ST, BCL11B, JUNB, TNFRSF4) and proteosome activation (PCBP2, PSMA6, PSMB10 and PSMA2). In contrast, the small number of genes that were up-regulated in CD4^+^ T cells appear to function to reduce NF-kB signaling (AES), proteosome activation (RARRES3) and apoptosis (MAL), again indicating a less activated state. Furthermore, as shown in **Figure 3G**, we found that in memory CD4^+^ T cells nasal Foralumab induced CTLA4 (T2, T3), KLRG1 (T4), and TGFB1 (T2). These results with KLRG1 are consistent with changes we observed following *in vitro* stimulation of CD4^+^ T cells by Foralumab (**Figure 1**).

The scRNA-Seq analysis of monocytes (**Figure 3E**) generated gene based clusters representing three classes of monocytes: 1) classical monocytes, which are associated with anti-bacterial activities; 2) non-classical monocytes, which are involved in immune surveillance, and 3) intermediate monocytes that are the most potent inducers of T cell activation. In the classical monocytes, the DEG genes with reduced expression are either induced by inflammation (LGALS1, SOD2, and CRIP1) or promote inflammation (GADD45B, DUSP1, FOS and SRGN). Of the DEG genes in the intermediate monocytes, five were genes that affect antigen presentation (HLA-DQB, HLA-DRB, CD74) or reduce the monocyte activation state (EFHD2 and RARRES3). In the non-classical monocytes, genes involved in antigen presentation (HLA-DPA1 and HLA-DPB1) were increased. It has been reported that DQ and DP restricted T cells produce higher levels of IL-10 whereas DR restricted T cells produce higher levels of IFNg (34). Thus, nasal Foralumab induces monocytes that promote a less inflammatory immune response.

In the Treg population (**Supplementary Figure 5**), four DEGs were identified, and all had decreased expression. Tregs had reduced expression of JUNB which may enhance Treg stability by inhibiting Th17 differentiation; USP15 which may reduce sensitivity to TGFb signaling, and MTRNR2L8 which may alter sensitivity to apoptosis.

We then examined the relationship of the identified differentially expressed genes to immune functions to determine whether the up or downregulated genes tended to be associated with a pro- or anti-inflammatory response. As shown in **Supplementary Figure 6** for CD8+ TEMRA cells, 17/19 genes that promoted inflammatory immune function were down-regulated, whereas 14/24 genes that dampened inflammatory immune function were up-regulated.

### Antigen microarrays

**Figure 4:**
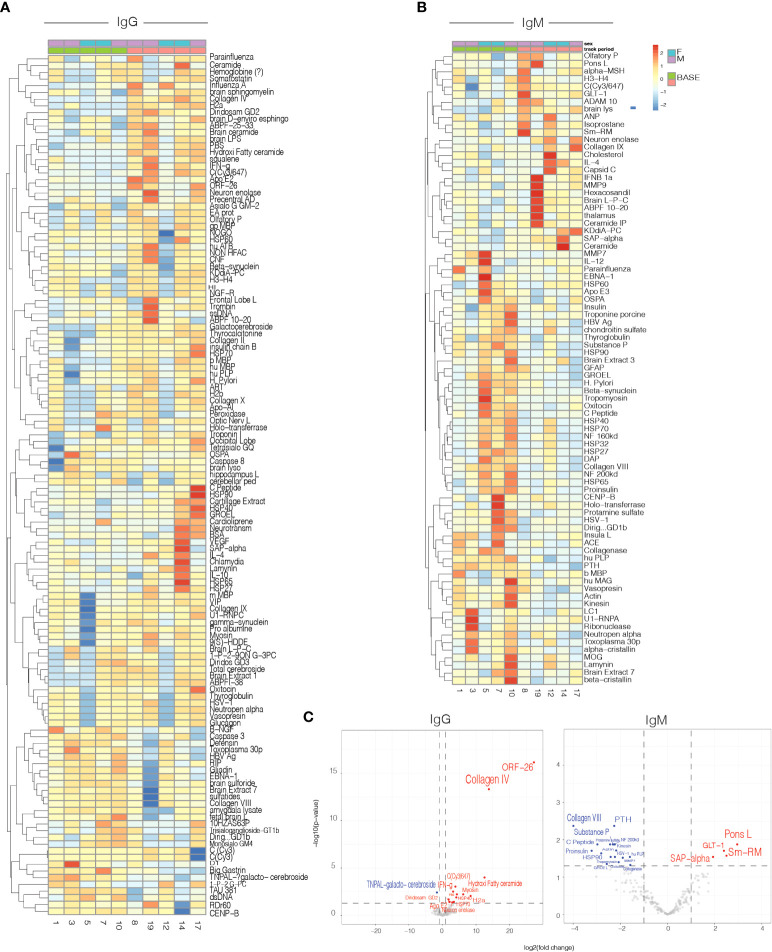
Serum IgG and IgM antibody reactivity in patients treated with 50ug. Heatmap representing the mean delta change from Timepoint 1(baseline) to Timepoint 2 (day 7) for: IgG **(A)** and IgM **(B)** antibody reactivity in serum samples from patients. **(C)** Volcano plot representing differential IgG and IgM antibody reactivity. Cut-off criteria was defined as p-value < 0.05 and log2 fold change > 1 or < −1.

Antigen microarrays are a unique tool for the study of the immune system in health (35) and disease (36, 37). We used an antigen microarray containing a broad panel of antigens (n=550) that included self and non-self-proteins, heat shock proteins, and infectious agents to investigate the effects of nasal Foralumab on the immune repertoire. We had previously used antigen arrays to investigate the immune response in healthy subjects treated with oral OKT3 antibody (19) **Figure 4A-C**. We measured the effect of nasal Foralumab on IgG and IgM reactivities determined at T1 vs T2 and found that changes were observed primarily in those receiving the 50ug dose (**Figures 4A–C**). **Figure 4A** shows that treatment with nasal Foralumab resulted in significant changes in the reactivity of the T-cell-dependent IgG repertoire. These findings are in agreement with the significant effects on the T cell response we detected in functional assays and by scRNAseq.

## Discussion

We found that nasal Foralumab given to healthy subjects was safe at doses of 10ug, 50ug and 250ug given for 5 consecutive days and that immune effects were predominantly observed at the 50ug dose. A dose effect with 50ug being more immunomodulatory than 250ug is consistent with animal studies of mucosal tolerance in which higher doses do not induce immune regulation, most likely due to the partial signaling that occurs at intermediate doses which favors the induction of regulatory cells (11, 25). The biologic effect of nasal anti-CD3 is markedly different from that which occurs with IV anti-CD3. IV anti-CD3 is associated with modulation of CD3 from the cell surface, a decrease in CD3 cells and side effects that include cytokine release syndrome and in some instances activation of EBV (1). EBV reactivation was observed with IV Foralumab at the 500ug and 1000ug doses. With nasal Foralumab, we did not observe EBV activation at any of the doses or modulation of CD3 from the cell surface and we did not find Foralumab in the bloodstream. Thus, unlike IV administered anti-CD3 which acts systemically by lysing CD3^+^ T cells, followed by immune reconstitution, nasal anti-CD3 acts locally at the mucosal surface as an immunomodulatory agent. In animal studies, we found nasal anti-CD3 localized to the cervical lymph nodes and as with human studies, we did not observe nasal anti-CD3 in the bloodstream of animals.

We compared the *in vitro* activation properties of Foralumab to a commonly used anti-CD3 monoclonal antibody UCHT1. We found that Foralumab induced preferential CD8^+^ T cell proliferation and reduced CD4^+^ T cell proliferation. Foralumab stimulation of purified CD4^+^ T cells resulted in higher expression of KLRG1, something we also observed in CD4 memory cells in the nasal Foralumab treated subjects. The *in vitro* assays also demonstrated that Foralumab-stimulated CD8 T cells exhibited regulatory function *via* their capacity to kill CD4 T cells.

In our animal studies, we found that nasal anti-CD3 induced LAP^+^ IL-10 secreting Tregs that could adoptively transfer protection (11, 12, 14, 15). We did not find a prominent increase in IL-10 in our human studies, though we did observe an increase of DN LAP^+^ T cells in 50ug treated subjects at the T4 timepoint, and LAP expression on CD4 and CD8 T cells after *in vitro* Foralumab stimulation. The major effects we observed with nasal Foralumab occurred in CD8^+^ T cells which is consistent with the effects observed with other anti-CD3 monoclonal antibodies given IV in humans (23, 32). We found a reduction of CD8^+^ effector memory cells, an increase in naïve CD8^+^ as well as CD4^+^ cells, and a reduction of CD8+ T cell granzyme B and perforin expression. In addition, our antigen array studies also showed most prominent effects at the 50ug dose.

scRNAseq analysis of the subjects receiving the 50ug dose allowed a more detailed analysis of the immune effects of nasal Foralumab. Although some of the DEGs functioned in homeostatic cell biologic processes, most of the affected DEGs had immunologic functions. In the CD8^+^ population the majority of the induced genes were anti-inflammatory. Interestingly, both *in vitro* stimulation and *in vivo* administration of Foralumab induced CD8 T cell expression of TIGIT and KLRG1 which was also observed with IV administration of Teplizumab (32). Furthermore, similar to the recent report that KIR^+^CD8^+^ T cells are regulatory *via* their capacity to suppress pathogenic T cells and are active in autoimmune diseases and COVID-19 (33), we found that the nasal Foralumab treated CD8^+^ TEMRA show induction of KIR3DL2 in addition to TIGIT, KLRG1 and TGFB1, while LAP (TGFb), TIGIT and KLRG1 are induced on *in vitro* Foralumab-stimulated CD8 T cells. As regulatory CD8 T cells are poorly defined (38) it is unclear whether the expression of these inhibitory molecules may define a regulatory CD8 T cell population. Similar patterns were observed in non-regulatory CD4^+^ T cells with downregulation of DEGs associated with activated subsets. Upregulated genes in CD4^+^ memory cells included KLRG1 and TGFB1, which is consistent with what we observed following *in vitro* stimulation of CD4 T cells by Foralumab. Only minimal changes were observed in the Treg population with only 4 DEGs identified that included reduced expression of JUNB which may enhance Treg stability by inhibiting Th17 differentiation. Thus, it does not appear that nasal Foralumab is directly expanding classical Tregs. Changes were also observed in monocyte populations including expression of DQ and DP which are associated with T cells that produce higher levels of IL-10. Taken together, we found that nasal anti-CD3 has a strong immunomodulatory effect on the immune response that is dose dependent, decreases inflammation and promotes regulation.

Of note, as we were completing the present study of Foralumab in healthy volunteers, the scientific community was confronted with the COVID pandemic and the need for therapeutic approaches to treat the immune hyperactivity that occurs. We thus performed a pilot study in which 39 mild to moderate COVID-19 patients were given nasal Foralumab at a dose of 100 µg daily for 10 days or placebo (39). We observed a reduction of serum IL-6 and C-reactive protein in Foralumab treated subjects and more rapid clearance of lung infiltrates. Foralumab treatment was well-tolerated with no severe adverse events. These results further establish the safety and immune modulatory properties of Foralumab. We are currently undertaking scRNAseq analysis of this COVID treated cohort.

In the current study we treated subjects for 5 consecutive days and measured immune responses over the ensuing month. Marked changes were observed at the 50ug dose beginning at day 7 following treatment, after which the response began to wane. An important question relates to how frequently subjects with disease should be treated and with what regimen. To this end, we have initiated treatment of a subject with non-active progressive MS in which nasal Foralumab was given at a dose of 50ug three times per week in cycles involving 2 weeks on therapy followed by one week off therapy. We found that nasal Foralumab in this non-active SPMS patient treated over a 12-month period reduced microglial activation on [F-18]PBR06 PET imaging, decreased levels of proinflammatory cytokines, and had positive clinical effects. No side effects were observed (40). Additional patients are currently being treated with nasal Foralumab at the Brigham MS Center.

In summary, we found that nasal Foralumab is safe and induces immune effects at a dose of 50ug given for 5 consecutive days. Nasal Foralumab is a novel form of immunotherapy therapy for the treatment of patients with progressive neurologic disease in which microglial activation occurs including MS and Alzheimer’s disease.

## Data availability statement

The datasets presented in this study can be found in GEO online repository as GSE217357 and can be found at https://www.ncbi.nlm.nih.gov/geo/query/acc.cgi?acc=GSE217357.

## Ethics statement

The studies involving human participants were reviewed and approved by Mass General Brigham Human Subjects Research Committee (IRB). The patients/participants provided their written informed consent to participate in this study.

## Author contributions

TC designed the study with CMB-A, led and monitored this clinical study and wrote the manuscript; BK, JC, KH, ZheL, and JV performed immunologic studies; BH statistical analysis; CG bioinformatics; TS project management; SS immunologic studies; HL bioinformatics; TGM wrote manuscript; JZ clinical monitoring; RR and RB ENT evaluations; FG, MT, ZhaL, and FQ antigen arrays; WC provided input regarding dose and device selection, study design and adverse event monitoring, KS provided input regarding drug use and adverse event monitoring; HW designed the study and wrote the manuscript; CMB-A designed and performed the immunologic studies and scRNA-Seq analyses, and wrote the manuscript. All authors contributed to the article and approved the submitted version.

## Funding

This work was supported by the Ann Romney Center for Neurologic Diseases. The resources provided by the Center for Clinical Investigation at Brigham and Women’s Hospital were supported by grant 1UL1TR002541-01.

## Acknowledgments

We thank the BWH flow cytometry and sequency core facility members Rajesh Kumar, Adam Chicoine, Kevin Wei, and Zhu Zhu. We thank all subjects that voluntarily participated in this study. Our special thanks to Kevin Zinchuk, ParmD and the Investigational Drug Services pharmacy team, and the nursing team and staff at the Clinical Center for Clinical Investigation at the Brigham and Women’s Hospital. We thank the physicians of the Otolaryngology Division for for ENT monitoring. We thank the Tiziana Life Science team for their assistance with drug administration during this study.

## Conflict of interest

WC was employed by Clementi, Ltd. HLW is chair of the scientific advisory board of Tiziana Life Sciences and received consulting fees and stock options from the company. KS is an employee of Tiziana Life Sciences. TC is a member of the scientific advisory board and serves as a consultant to Tiziana Life Sciences. CMB-A serves as a consultant to Tiziana Life Sciences.

The remaining authors declare that the research was conducted in the absence of any commercial or financial relationships that could be construed as a potential conflict of interest.

## Publisher’s note

All claims expressed in this article are solely those of the authors and do not necessarily represent those of their affiliated organizations, or those of the publisher, the editors and the reviewers. Any product that may be evaluated in this article, or claim that may be made by its manufacturer, is not guaranteed or endorsed by the publisher.
