# Supplementary material for: Nasal administration of anti-CD3 monoclonal antibody modulates effector CD8+ T cell function and induces a regulatory response in T cells in human subjects
Source: Front Immunol. 2022 Nov 23;13:956907. doi: 10.3389/fimmu.2022.956907 (PMC9727230; doi:10.3389/fimmu.2022.956907)
Supplement: Supplementary file 1 [file DataSheet_1.pdf]

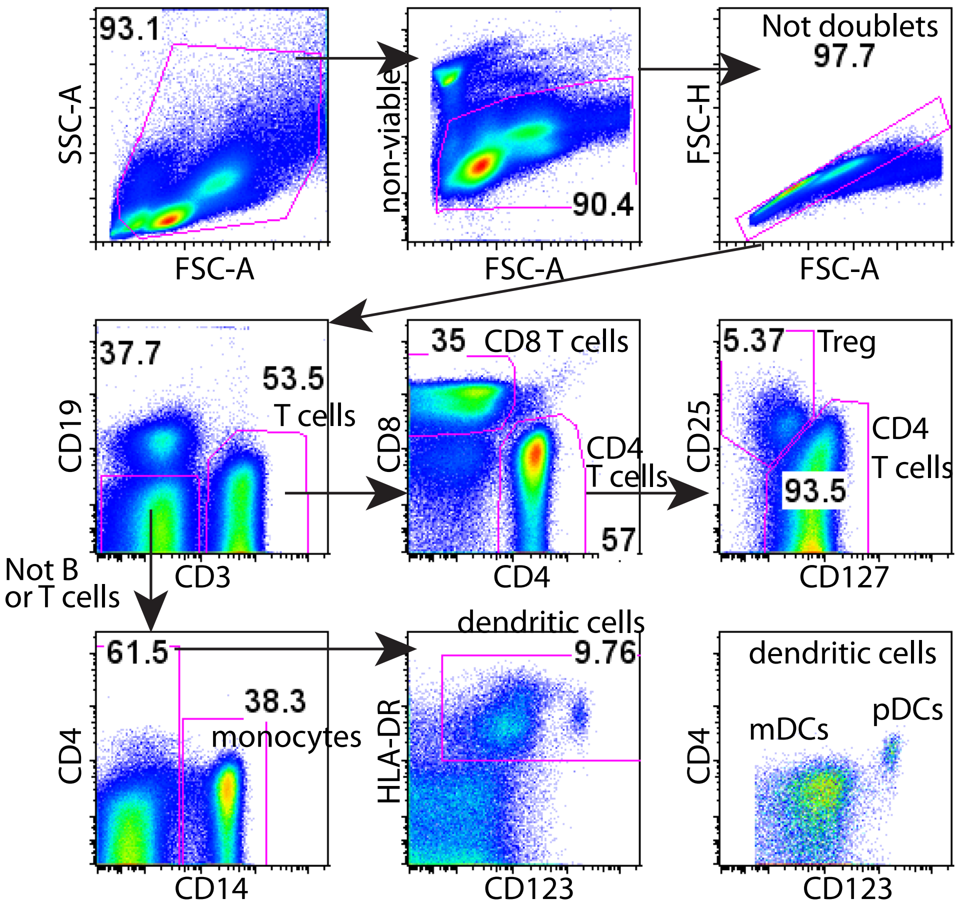

Stain	clone	Source
e506 viability dye		Thermo Fisher Scientific
CD19 APC/Fire 750	H1B19	BioLegend
CD3 APC	OKT3	BioLegend
CD8 AF700	L307.4	Becton Dickenson
CD4 BV785	OKT4	BioLegend
CD25 PE	BC96	BioLegend
CD127 Pacific Blue	A019D5	BioLegend
CD14 BV650	M5E2	BioLegend
HLA-DR PerCPCy5.5	L243	BioLgend
CD123 PeCy7	6H6	BioLegend

Supplementary Figure 1. FACS gating for isolation of the specific immune populations examined by single cell RNA-Seq by 10X genomics. Longitudinal PBMCs from T1-T4 after Foralumab nasal treatment (50ug/ml dose) were stained with e506, FcR block, and then with the indicated Mabs to allow isolation of viable subsets of CD8+ T cells, non-regulatory CD4+ T cells (not CD25hiCD127low), CD4+ Tregs (CD25hiCD127low), total monocytes, and total DCs. The samples were hash-tagged and each scRNA-Seq samples was generated with the same type of sorted immune population.
