# Supplementary figures and images for: Nasal administration of anti-CD3 monoclonal antibody modulates effector CD8+ T cell function and induces a regulatory response in T cells in human subjects
Source: Front Immunol. 2022 Nov 23;13:956907. doi: 10.3389/fimmu.2022.956907 (PMC9727230; doi:10.3389/fimmu.2022.956907)

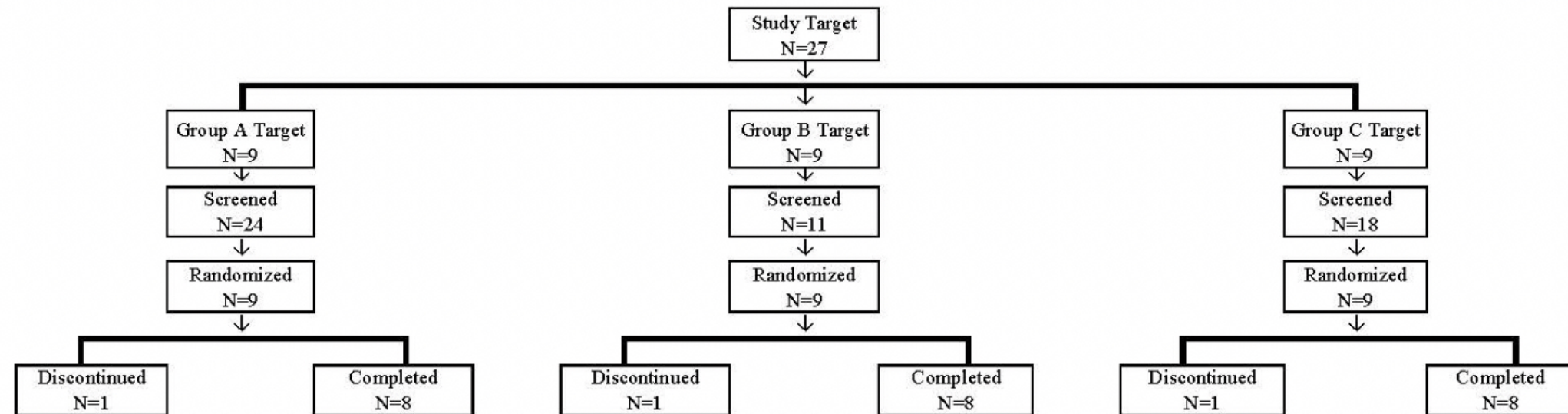

**Supplementary Figure 2.** Patient disposition.

Supplement: Supplementary file 2 [file DataSheet_2.pdf]
