# Supplementary material for: Nasal administration of anti-CD3 monoclonal antibody modulates effector CD8+ T cell function and induces a regulatory response in T cells in human subjects
Source: Front Immunol. 2022 Nov 23;13:956907. doi: 10.3389/fimmu.2022.956907 (PMC9727230; doi:10.3389/fimmu.2022.956907)
Supplement: Supplementary file 3 [file DataSheet_3.pdf]

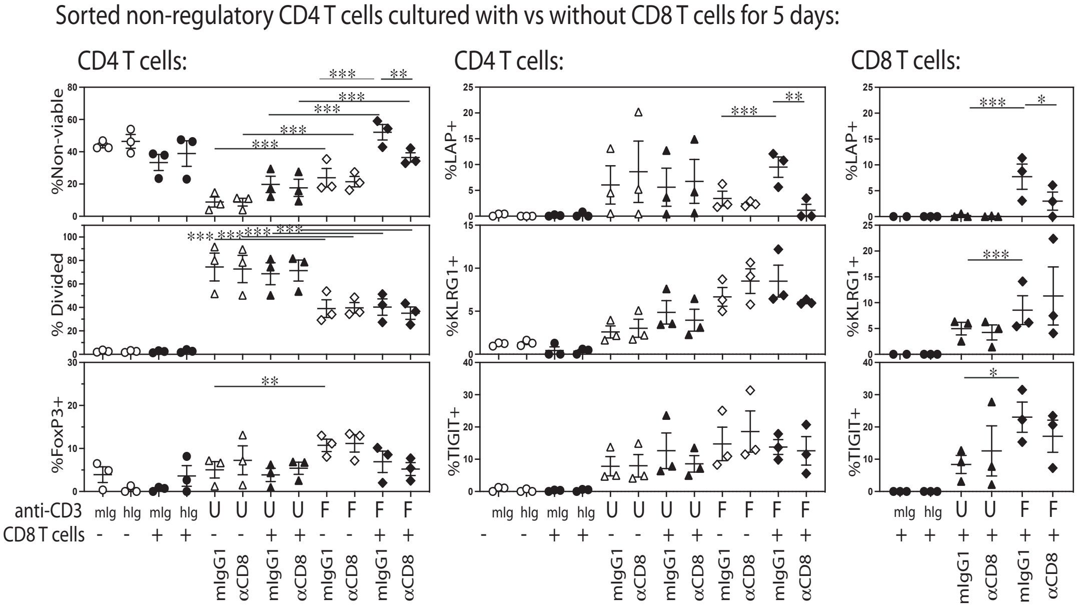

Supplementary Figure 3. The removal of regulatory T cells from the CD4+ T cell populations cultured alone or with autologous CD8 T cells has no effect on Foralumab stimulation of CD4+ T cells and the effects of CD8+ co-culture. CD4+ T cells that were FACS sorted to specifically not include CD25hiCD127lo Tregs were stimulated with and without autologous FACS sorted CD8+ T cells as shown in figure 1D. All features of the CD4+ or CD4+ and CD8+ co-cultures were identical to the cultures established with total CD4 T cells with the exception that the level of Foralumab induced FoxP3 was reduced in Treg depleted CD4+ T cells. Here we tested FACS-sorted CD4 T cells that were specifically gated to not include CD25hiCD127low Tregs, and stimulated them alone (open) or together with autologous FACS-sorted CD8 T cells (filled) with mIgG1 (isotype for UCHT1), huIgG1 (isotype for Foralumab), UCHT1 or Foralumab. All in the presence of IL-2 (5U/ml), and T cell depleted irradiated PBMCS as APCs. On day 5, the cultures were harvested and measured for viability, proliferation, and surface expression of LAP, KLRG1 and TIGIT, and intracellular FoxP3. Significance was determined by One-Way ANOVA with Sidak's correction for multiple comparisons, * $p<0.05$, ** $p<0.01$, *** $p<0.005$.
