# Supplementary material for: Nasal administration of anti-CD3 monoclonal antibody modulates effector CD8+ T cell function and induces a regulatory response in T cells in human subjects
Source: Front Immunol. 2022 Nov 23;13:956907. doi: 10.3389/fimmu.2022.956907 (PMC9727230; doi:10.3389/fimmu.2022.956907)
Supplement: Supplementary file 4 [file DataSheet_4.pdf]

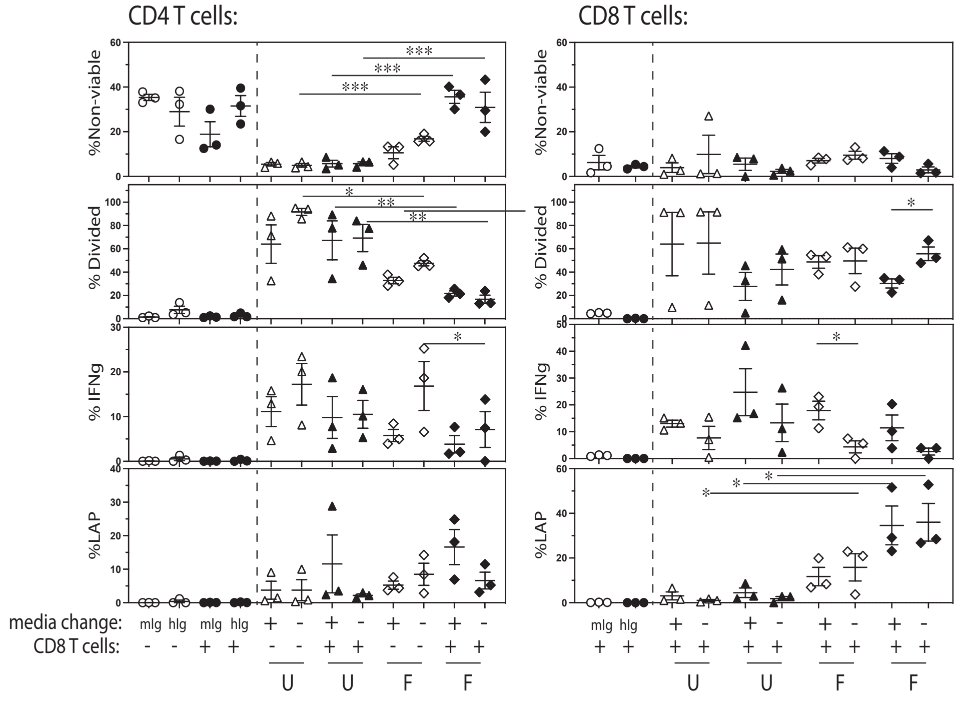

Supplementary Figure 4. Replacing media at day 3 to avert nutrient exhaustion does not change the unique properties of in vitro Foralumab stimulation. FACS sorted total CD4+ and total CD8+ T cells were each plated alone or together (at 2:1 ratio) in cultures stimulated with UCHT1/IL-2 or Foralumab /IL-2 and CD3-depleted APCs. On day 3 of incubation, half of the media (100ul) was replaced with fresh media containing 5U IL-2 in one set of replica plates. On day 5, the cultures that had media replaced and the control untouched cultures were harvested and stained as described and the features of the CD4+ T cells (left) or CD8+ T cells (right) are shown. Filled symbols are the cells that were stimulated in the presence of the other cell type, the open symbols are the cells that were stimulated alone. Significance was determined by One-Way ANOVA with Sidak's correction for multiple comparisons, * p<0.05, **p<0.01, ***p<0.005.
