# Supplementary material for: Nasal administration of anti-CD3 monoclonal antibody modulates effector CD8+ T cell function and induces a regulatory response in T cells in human subjects
Source: Front Immunol. 2022 Nov 23;13:956907. doi: 10.3389/fimmu.2022.956907 (PMC9727230; doi:10.3389/fimmu.2022.956907)
Supplement: Supplementary file 5 [file DataSheet_5.pdf]

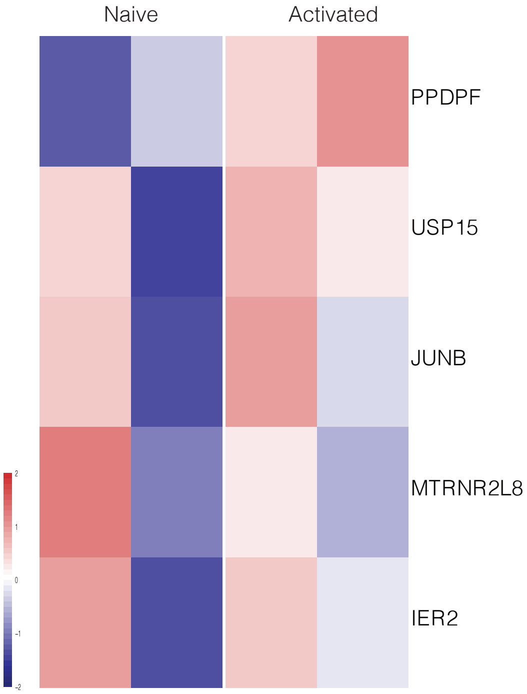

Supplementary Figure 5. Treg heat map. The DEG found between baseline (T1) and T2 in CD4+CD25^{hi} CD127^{lo} Tregs are shown for naïve Tregs and activated (memory) Tregs.
