# Supplementary material for: Nasal administration of anti-CD3 monoclonal antibody modulates effector CD8+ T cell function and induces a regulatory response in T cells in human subjects
Source: Front Immunol. 2022 Nov 23;13:956907. doi: 10.3389/fimmu.2022.956907 (PMC9727230; doi:10.3389/fimmu.2022.956907)
Supplement: Supplementary file 6 [file DataSheet_6.pdf]

Relationship of differentially expressed genes in CD8+ TEMRA to immune function

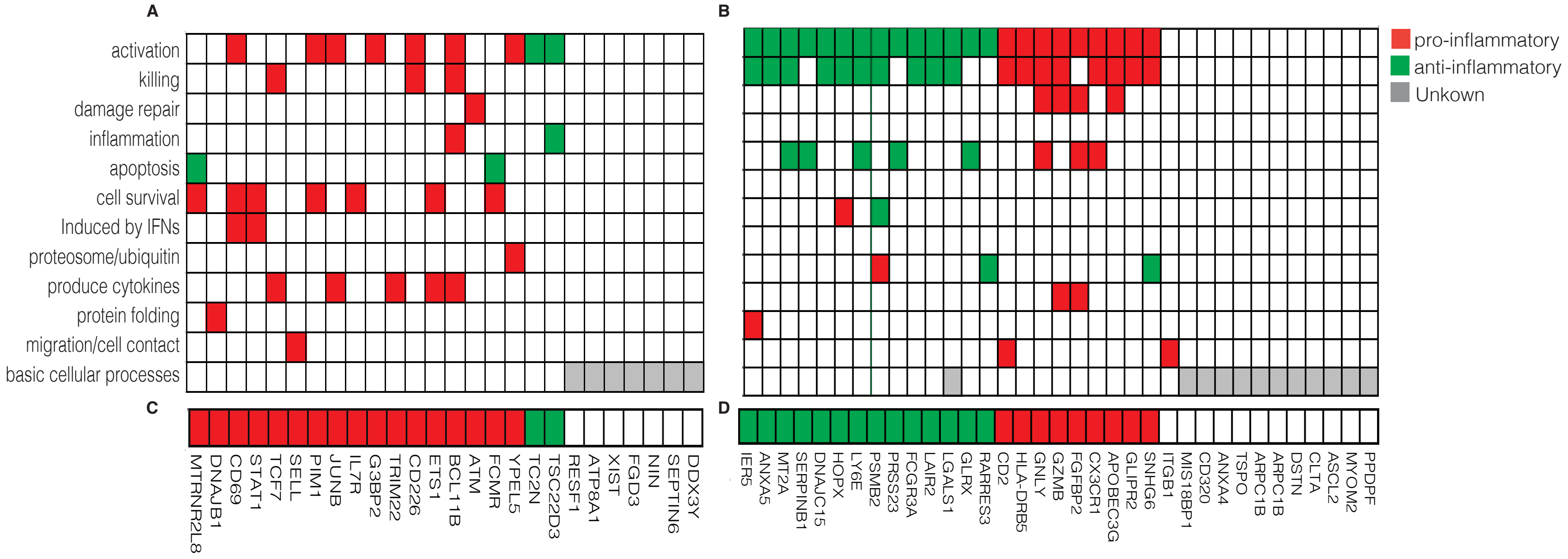

Supplementary Figure 6. Relationship of differentially expressed genes in CD8+ TEMRA to immune function. A, B). The functional properties of the DEGs identified were sourced from the literature and assigned to one of twelve groups C) Genes that were downregulated from T1 to T2 after treatment. D) Genes that were upregulated from T1 to T2 after treatment. Red indicates a pro inflammatory role for a specific immune function. Green indicates an anti-inflammatory role for a specific immune function. As shown in C, 17/19 genes with a pro inflammatory role were downregulated with treatment whereas in D, 16/24 genes with an anti-inflammatory role were upregulated.
